# 25 years of *Drosophila* “Sleep genes”

**DOI:** 10.1080/19336934.2025.2502180

**Published:** 2025-05-06

**Authors:** Orie Thomas Shafer

**Affiliations:** Gill Institute for Neuroscience and Department of Biology, Indiana University in Bloomington, Bloomington, IN, USA

**Keywords:** Sleep, homeostasis, *Drosophila*, forward genetic screens, reverse genetics

## Abstract

The field of *Drosophila* sleep research, which began 25 years ago, has identified more than 200 genes influencing sleep. In this review, I summarize the foundation of the field and the growing list of genes implicated in sleep regulation. I compare the genetic methods used to identify genes governing sleep and circadian rhythms and the distinct outcomes of screens for genes regulating these two highly related processes. Finally, I discuss the ~ 200 sleep-regulating genes of *Drosophila* in the context of recent developments in the field and voice reasons for scepticism regarding the relevance of these genes to the homoeostatic regulation of sleep. Finally, I speculate on the future promise of the fly model system for revealing conserved molecular mechanisms of sleep homoeostasis.

## Sleep and its importance

Sleep is a basic biological need, and sufficient sleep is required for physical and mental health. Reduced sleep contributes to various metabolic, inflammatory, cardiovascular, and neurological disorders, reduces productivity and performance, and threatens public safety [1]. The widespread and negative consequences of sleep loss are so profound that the World Health Organization declared a ‘Global Epidemic of Sleeplessness’ in 2012 [2], and, according to the CDC, human sleep has not improved since. Understanding sleep regulation is, therefore, critical for health and society.

Sleep is primarily regulated by a circadian clock and a sleep homeostat, which promotes increasing sleep pressure during wakefulness, eventually forcing a transition from wakefulness to sleep. During sleep, this pressure falls until it reaches sufficiently low levels, resulting in the transition from sleep to waking [3]. The circadian clock modulates the amount of sleep pressure necessary to induce sleep/wake transitions across the day [3]. A ‘two-process’ model based on human circadian timekeeping and sleep homoeostasis produces remarkably accurate predictions for sleep duration and timing under both normal and sleep-deprived conditions [3]. Despite the central importance of homoeostatic sleep regulation, its underlying mechanisms are poorly understood. Even the best-characterized factors hypothesized to mediate sleep pressure do not fully account for the daily rise and fall of sleep pressure during regular sleep/wake cycles [4]. Thus, understanding the mechanisms underlying sleep homoeostasis is a central unmet goal of sleep science. *Drosophila melanogaster* has become a widely used genetic model for understanding sleep regulation [5]. Here, I summarize work done on this fly over the last quarter century to identify genes governing sleep.

## A quarter century of Drosophila sleep

Sleep-like states were first established for *Drosophila melanogaster* 25 years ago [6,7], and the species has since become a valuable and widely used model system for understanding sleep regulation [5]. The establishment of sleep in the fly was built upon the foundation of earlier work on other arthropod species. The earliest work on daily rhythms in flies was predominantly conducted on the rhythm of adult emergence in Drosophilid flies [8–10]. Taylor and Kalmus reported daily rhythms in flight and rest in 1954, describing a crepuscular flight rhythm in *Drosophila subobscura*, with relatively brief peaks at dawn and dusk separated by long bouts of relative inactivity [11]. This bimodal daily pattern of activity and rest is highly reminiscent of the now familiar daily locomotor activity rhythms of *Drosophila melanogaster* [12]. Though Taylor and Kalmus did not speculate on the relationship between *subobscura*’s two daily peaks of rest and sleep in mammals, theirs was the first observation of what we would now recognize as two daily windows of sleep in *Drosophila*.

Though a sleep-like state had been described in the Mediterranean flour moth (*Anagasta kuehniella*) as early as 1968 [13], the establishment of sleep-like states in invertebrates was led by Irene Tobler, who, based on the work of Piéron [14], summarized four behavioural criteria for the identification of sleep like states. These were quiescence, the assumption of a characteristic posture, elevated arousal threshold, and rapid reversibility given sufficient stimulation [15]. Tobler suggested the addition of a fifth possible criterion for identifying sleep-like states akin to mammalian sleep: homoeostatic control, proposing that a behavioural state akin to mammalian sleep should display a rebound after depriving an animal of that state [15]. Using these criteria, Tobler identified sleep-like states in cockroaches [16,17] and scorpions [18].

In the early 2000s, two studies established that rest in *Drosophila melanogaster* met Tobler’s criteria [6,7]. This work independently established the presence of periods of inactivity accompanied by a decrease in sensitivity to mechanical stimulation [6,7]. Shaw and colleagues (2000) also showed that quiescence was rapidly reversible upon delivery of sufficiently strong vibrational stimuli [7]. Both studies also showed depriving flies of this quiescent state produced subsequent increases in rest and that caffeine reduced rest in flies [6,7]. Shaw and colleagues reported that blocking histamine receptors increased rest in flies [7]. These results indicated that substances known to increase and decrease sleep in mammals had similar effects in *Drosophila* [6,7]. Hendricks and colleagues (2000) also provided evidence that flies take up specific postures and positions relative to food during rest [6]. Together, these two studies firmly established a sleep-like state in flies, now widely considered sleep.

The establishment of fly sleep was highly significant given the well-established utility of forward genetic screens in *Drosophila* [19]. A major strength of such screens is that they are entirely unbiased, requiring no prior knowledge regarding the molecular and cellular underpinnings of the biological process one seeks to understand. Such screens had already uncovered highly conserved genes regulating development and behaviour when fly sleep was first established. For example, screens successfully identified genes involved in properly developing the segmental body plan (e.g [20,21]), and behavioural circadian timekeeping (e.g [22,23]). In the case of screens for larval cuticle patterning and adult circadian timekeeping, forward genetic screens approached saturation; that is, they successfully identified nearly all the genetic loci that could produce a phenotype when mutated, as evidenced by repeated isolation of new alleles of the same loci [21,24]. Remarkably, for circadian clock screens, these amounted to only approximately a dozen genes whose gene products were subsequently shown to interact within the very same cellular mechanism to create daily rhythms in gene expression [25]. With the establishment of fly sleep, it was reasonable for confidence to be high that genetic screens in *Drosophila* would reveal the genetic and, based on this, the cellular basis of sleep regulation [26].

Effective genetic screens must allow for the examination of many potential mutant lines. For this reason, they must be built upon simple methods to detect phenotypes with high-throughput assays. Forward genetic screens for sleep mutants adopted a methodology first developed for the circadian field that allowed daily sleep to be measured for thousands of potential mutants: the Trikinetics Drosophila Activity Monitors (DAMs). DAMs, which track infrared beam crossings by flies housed in single, glass capillary tubes as a proxy for activity, had been used successfully by multiple research groups in screens for circadian rhythms mutants [27]. The field defined sleep as any period of inactivity (i.e. the absence of a single beam crossing) that was five minutes or longer [7,28].

In (2003), Chiara Cirelli published a report on an ongoing large-scale forward genetic screen for sleep mutants that involved screening ~ 2000 chemically mutagenized fly lines and ~ 3000 lines bearing P-element insertions as potential disruptors of gene function [26]. At the time of this publication, the examination of these ~ 5000 lines had yielded 10 lines displaying significantly reduced daily sleep [26]. The genes affected by two of these mutations were identified in subsequent publications as *Shaker*, the alpha subunit of a voltage-gated potassium channel [29], and *Hyperkinetic*, the beta subunit of the same channel [30]. Another screen of ~ 3500 transposon insertions for short sleeping mutants conducted in the laboratory of Amita Sehgal identified *sleepless* (a.k.a. *quiver*), a membrane protein required for *Shaker* function, as a gene required for normal levels of sleep [31]. Remarkably, an independent screen of over 3000 potential mutants in the lab of Michael Young revealed two additional alleles of *Shaker* [32]. The convergent identification of *Shaker* in independent screens and the identification of the *Shaker*-related *Hyperkinetic* and *sleepless/quiver* was strong evidence that a specific voltage-gated potassium channel played a significant role in sleep regulation in *Drosophila.*

Contemporaneously with the first forward genetic screens for sleep mutants, a short sleeping mutant was serendipitously discovered in the laboratory of F. Rob Jackson. This mutant, named *fumin*, displayed less than half the sleep of normal flies, an effect caused by disrupting the fly homolog of the mammalian dopamine transporter [33]. A screen of chemically mutagenized flies was conducted in the laboratory of Amita Sehgal that isolated another allele of *fumin*, which displayed about a two-thirds reduction in sleep relative to normal flies [34]. Thus, early and independent work converged on dopamine signalling as a regulator of sleep fly sleep.

Stavropoulos and Young reported a screen of ~ 3,500 lines for reduced sleep phenotypes and identified *insomniac* as a gene required for the proper amount of daily sleep [32]. Severely hypomorphic alleles of the *insomniac* gene, which encodes an adaptor protein involved in protein degradation, resulted in a ~ 75% sleep loss [32]. Additional screens in the lab of Amita Sehgal identified *Redeye* [35], which encodes nicotinic acetylcholine receptor alpha subunit, and *argus* [36], a regulator of autophagy, as regulators of baseline sleep amount. Afonso and colleagues identified *Taranis*, a cell cycle regulator, in a forward genetic screen [37]. Finally, Liu and colleagues used a baseline sleep screen to identify *wide awake*, which encodes a regulator of GABA receptor expression [38]. Dubowy et al. (2016) conducted an alternative unbiased screening approach to isolate mutants that displayed abnormal sleep rebound following sleep deprivation [39]. However, the two mutants identified in this screen were not mapped unequivocally to specific genes. Thus, forward genetic screens for daily sleep amount have identified nine sleep-regulating genes, which are involved in a few general mechanisms of sleep regulation: control of neuronal firing through direct effects on neuronal signalling (*Shaker, Hyperkinetic, fumin, sleepless/quiver*, *Redeye*, and *wide awake*) or the cellular homoeostasis pathways of protein degradation (*insomniac*), autophagy (*argus*), and cell cycle regulation (*taranis*). Table 1 summarizes the ‘sleep genes’ identified by forward genetic screens.

**Table 1.** Sleep regulating genes identified in unbiased genetic screens. Note: *fumin* was first discovered serendipitously in the lab of F. Rob Jackson but then identified independently in a forward genetic screen in the lab of Amita Sehgal. ND (Not done) indicates instances where rebound following sleep deprivation has not been reported. For brevity/clarity, studies are cited using PubMed ID (PMID). ‘General mechanism’ and ‘Specific mechanism’ are my attempts to indicate the molecular/cellular function of the sleep gene product. The author acknowledges that there are likely other, equally good, and possibly better ways to categorize genes.

Gene Name	Role in Baseline Sleep	Role in Sleep Rebound	Year	PMID	General Mechanism	Specific Mechanism
Shaker	Promotes Sleep	None	2005, 2011	15858564, 22196332	Neural Signalling	Potassium Channel
fumin	Promotes Sleep	Promotes Rebound Following Deprivation	2005, 2008	16093388, 18457233	Neural Signalling	Aminergic Signalling
Hyperkinetic	Promotes Sleep	None	2007	17507560	Neural Signalling	Potassium Channel
sleepless (a.k.a., quiver)	Promotes Sleep	Promotes Rebound Following Deprivation	2008	18635795	Neural Signalling	Potassium Channel
insomniac	Promotes Sleep	Promotes Rebound Following Deprivation	2011	22196332, 23055946	Cellular Homoeostasis	Protein Degradation
Redeye	Promotes Sleep	ND	2014	24497543	Neural Signalling	Cholinergic Signalling
wide awake	Promotes Sleep	ND	2014	24631345	Neural Signalling	GABAergic Signalling
taranis	Promotes Sleep	ND	2015	26096977	Cellular Homoeostasis	Cell Cycle Regulation
argus	Promotes Sleep	ND	2021	34085929	Cellular Homoeostasis	Autophagy

In addition to using forward genetic screens, investigators have examined mutants discovered by previous studies of other biological phenomena. Furthermore, the sequencing and annotation of the *Drosophila melanogaster* genome [40], which was first published in the same year as fly sleep was first defined, set the stage for using ‘reverse genetics’ to understand sleep. This approach targets specific genes for loss of function or overexpression [41]. Such a reverse genetics approach was used alongside the forward genetic screens described above. For example, Hendricks and colleagues (2001) established a role for cAMP signalling and the cAMP response element binding protein in sleep regulation by analysing previously described mutants and transgenes for the overexpression of cAMP signalling components [42].

One major approach to reverse genetics was the *Drosophila* gene disruption project, which established large libraries of fly stocks bearing mobile P-element insertions that can disrupt genes to produce mutations [43,44]. The first sleep screens used insertional mutations alongside chemical mutagenesis (e.g [26,31]. The gene disruption project also made a reverse genetic approach possible by allowing investigators to test mutants for conserved fly genes hypothesized to be involved in sleep regulation (e.g [45]. The establishment of genome-wide libraries of transgenes encoding elements for targeted RNA interference further expanded the reverse genetics toolkit [46] and has been used extensively in the fly sleep field to knock down identified genes within specific cell types. Reverse genetics, in addition to complementing forward genetic screens to identify sleep-regulating genes, also allowed investigators to confirm and characterize the function of genes identified with forward genetics (e.g [32,36]). Investigators have also incorporated CRISPR/Cas9 knock-outs as a reverse genetic approach to identify fly sleep regulators (e.g [36,47,48]).

**Figure 1.**
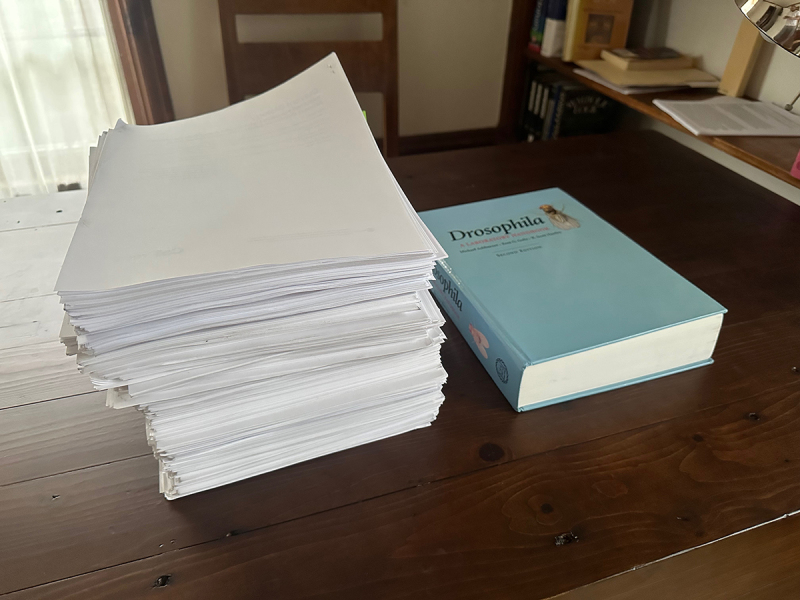
The printed results of a February 2024 PubMed search of ‘*drosophila* sleep’ in which the genes listed in Tables 1–3 were identified. M. Ashburner’s ‘*Drosophila*, a laboratory manual’, a 434-page volume, is shown for scale.

Though forward genetic screens identified a small number of sleep-regulating genes acting via a few cellular mechanisms, reverse genetics has implicated many more genes acting via diverse mechanisms. An examination of a Public Library of Medicine Search for ‘Drosophila Sleep’ conducted in February of 2024 revealed approximately 200 genes implicated in sleep regulation using reverse genetics (Figure 1; Table 2; Supplemental Table S1). These genes were identified by gene disruption, RNA interference knockdown, or, in the case of genes encoding micro-RNAs (miRNA), the expression of dominant negative mRNA ‘sponges’ containing multiple complementary sequences that result in miRNA loss of function.

**Table 2.** Sleep regulating genes identified using reverse genetic methods. See the legend for table 1 and the text for details regarding the organization, categorization, and omission of sleep-regulating genes.

Gene	Role in Baseline Sleep	Role in Sleep Rebound	Year	PMID	General Mechanism	Specific Mechanism
speck (a.k.a. Dat and AANAT1)	None	Suppresses Rebound Following Deprivation	2000, 2020	10710313, 32955431	Neural Signalling	Aminergic Signalling
cAMP Response Element Binding Protein B (CrebB)	Promotes Wakefulness	Suppresses Rebound Following Deprivation	2001	11687816	Second Messenger Signalling	cAMP Signalling
dunce	Promotes Sleep	ND	2001	11687816	Second Messenger Signalling	cAMP Signalling
rutabaga	Promotes Wakefulness	ND	2001	11687816	Second Messenger Signalling	cAMP Signalling
5-hydroxytryptamine (serotonin) receptor 1A	Promotes Sleep	None	2006, 2017	16753559, 28984573	Neural Signalling	Aminergic Signalling
Relish	Promotes Sleep	None	2007	17520783	Gene Regulation	Inflammation/Immunity
Heat shock protein 70 cognate 3 (a.k.a., BiP and Grp78)	None	Promotes Rebound Following Deprivation	2007	17552370	Cellular Homoeostasis	Unfolded Protein Response
rhomboid	Promotes Sleep	Promotes Rebound Following Deprivation	2007	17694052	Cellular Homoeostasis	Growth Factor Signalling
Resistant to dieldrin	Promotes Sleep	ND	2008	18223647, 19038223	Neural Signalling	GABAergic Signalling
Tyrosine decarboxylase 2	Promotes Wakefulness	ND	2008	18799671	Neural Signalling	Aminergic Signalling
Tyramine β hydroxylase	Promotes Wakefulness	ND	2008	18799671	Neural Signalling	Aminergic Signalling
Activating transcription factor-2	Promotes Sleep	ND	2008	18694958	Gene Regulation	Transcription Factor, Stress Response
Pigment dispersing factor	Promotes Wakefulness	ND	2008	19038223	Neural Signalling	Neuropeptide Signalling
Fragile X messenger ribonucleoprotein 1	Promotes Wakefulness	Promotes Rebound Following Deprivation	2009	19228950	Gene Regulation	RNA Translation and Trafficking, Neural Development and Plasticity
Dopamine 1-like receptor 1	Promotes Wakefulness	ND	2009	19945394	Neural Signalling	Aminergic Signalling
Ecdysone receptor	Promotes Sleep	Promotes Rebound Following Deprivation	2010	20215472	Gene Regulation	Nuclear Hormone Receptor, Steroid Signalling
brummer	None	Suppresses Rebound Following Deprivation	2010	20824166	Cellular Homoeostasis	Lipid metabolism and Storage
Lipid storage droplet-2	None	Promotes Rebound Following Deprivation	2010	20824166	Cellular Homoeostasis	Lipid metabolism and Storage
Elongator complex protein 3	Promotes Sleep	ND	2010	20626565	Gene Regulation	Chromatin Regulation
Angiotensin-converting enzyme-related	Promotes Sleep	ND	2011	21270318	Cellular Homoeostasis	Peptidase, Heart Rate Regulation, Aging
G protein alpha subunit o	Promotes Sleep	ND	2011	21358844	Cellular Homoeostasis	G Protein Signalling
bunched	None	Promotes Rebound Following Deprivation	2011	21549599	Gene Regulation	Transcription Factor
Notch	None	Suppresses Rebound Following Deprivation	2011	21549599	Gene Regulation	Cell Fate Regulation
sarah	Promotes Sleep	ND	2011	21900555	Cellular Homoeostasis	Calcium Signalling, Mieosis, Courtship Behavior
Calcineurin A at 14F	Promotes Sleep	None	2011	21900555, 21917797	Cellular Homoeostasis	Calcium Signalling
Calcineurin B	Promotes Sleep	None	2011	21900555, 21917797	Cellular Homoeostasis	Calcium Signalling
yellow-achaete intergenic RNA	Promotes Sleep	Promotes Rebound Following Deprivation	2011	21775470	Gene Regulation	Noncoding RNA
Cullin-3	Promotes Sleep	Promotes Rebound Following Deprivation	2011, 2012	22196332, 23055946	Cellular Homoeostasis	Protein Degradation
Nedd8 ubiquitin like modifier	Promotes Sleep	ND	2011	22196332	Cellular Homoeostasis	Protein Degradation
Homer	Promotes Sleep	None	2012, 2019	22532843, 31418019	Neural Signalling	Glutamatergic Signalling
insomniac	Promotes Sleep	Promotes Rebound Following Deprivation	2012	23055946	Cellular Homoeostasis	Protein Degradation
basket	Promotes Sleep	None	2011	22197814	Gene Regulation	Kinase Signalling, Cell shape Regulation, Stress Response
regulator of cyclin A1	Promotes Sleep	ND	2012	22461610	Cellular Homoeostasis	Cell Cycle Control
cyclin A	Promotes Sleep	Promotes Rebound Following Deprivation	2012	22461610	Cellular Homoeostasis	Cell Cycle Control
Sulfonylurea receptor	Promotes Sleep	ND	2013	22105623	Cellular Homoeostasis	Potasium Channel, Heart Development
Vesicular monoamine transporter	Promotes Wakefulness	None	2013	23658190	Neural Signalling	Aminergic Signalling
Histidine decarboxylase	Promotes Wakefulness	ND	2013	23844178	Neural Signalling	Histamine Signalling
Histamine-gated chloride channel subunit 1	Promotes Wakefulness	ND	2013	23844178	Neural Signalling	Histamine Signalling
short neuropeptide F receptor	Promotes Wakefulness	ND	2013	23796436	Neural Signalling	Neuropeptide Signalling
Neuroligin 4	Promotes Sleep	ND	2013	24068821	Neural Signalling	Cell Adhesion, Synaptic Development
SIFamide	Promotes Sleep	ND	2014	24658384	Neural Signalling	Neuropeptide Signalling
SIFamide receptor	Promotes Sleep	ND	2014	24658384	Neural Signalling	Neuropeptide Signalling
Sex peptide receptor	Promotes Sleep	Promotes Rebound Following Deprivation	2014	25333796	Neural Signalling	Neuropeptide Signalling
Myoinhibiting peptide precursor	Promotes Sleep	Promotes Rebound Following Deprivation	2014	25333796	Neural Signalling	Neuropeptide Signalling
Diuretic hormone 31	Promotes Wakefulness	ND	2014	25455031	Neural Signalling	Neuropeptide Signalling
γ-aminobutyric acid transaminase	Promotes Wakefulness	ND	2015	24637426	Neural Signalling	GABAergic Signalling
NMDA receptor 1	Promotes Sleep	ND	2015, 2019	26023770, 31064979	Neural Signalling	Glutamatergic Signalling
Insulin-like peptide 1	Promotes Sleep	ND	2015	25581915	Neural Signalling	Insulin Signalling
Insulin-like peptide 2	Promotes Sleep	ND	2015	25581915	Neural Signalling	Insulin Signalling
Insulin-like peptide 3	Promotes Sleep	ND	2015	25581915	Neural Signalling	Insulin Signalling
Insulin-like peptide 5	Promotes Sleep	ND	2015, 2021	25581915, 34998032	Neural Signalling	Insulin Signalling
Insulin-like peptide 6	Promotes Sleep	ND	2015	25581915	Neural Signalling	Insulin Signalling
Insulin-like peptide 7	Promotes Sleep	ND	2015	25581915	Neural Signalling	Insulin Signalling
Insulin-like receptor	Promotes Sleep	ND	2015	25581915	Neural Signalling	Insulin Signalling
Anaplastic lymphoma kinase	Promotes Wakefulness	None	2015	26536237	Neural Signalling	Receptor Tyrosine Kinase Signalling
Neurofibromin 1	Promotes Sleep	ND	2015, 2023	26536237, 37593040	Cellular Homoeostasis	G Protein Signalling
Ca2±channel protein ?1 subunit T	Promotes Wakefulness	ND	2015	26647714	Neural Signalling	Calcium Channel
Adenosine deaminase acting on RNA	Promotes Wakefulness	None	2016	26813350	Gene Regulation	RNA Editing
Dopamine 1-like receptor 2	Promotes Wakefulness	ND	2016	27487216	Neural Signalling	Aminergic Signalling
Shaker cognate b	Promotes Sleep	ND	2016	27487216	Neural Signalling	Potasium Channel
sandman	Promotes Wakefulness	ND	2016	27487216	Neural Signalling	Potasium Channel
Transcription factor AP-2	Promotes Sleep	ND	2016	27829368	Gene Regulation	Transcription Factor
Neurexin 1	Promotes Sleep	Promotes Rebound Following Deprivation	2016	27905548	Neural Signalling	Neuropeptide Signalling
Vesicular acetylcholine transporter	Promotes Sleep	ND	2016	27905548	Neural Signalling	Cholinergic Signalling
β-Amyloid precursor protein binding protein 1	Promotes Wakefulness	ND	2017	28314820	Neural Signalling	Post-Translational Protein Modification
Excitatory amino acid transporter 1	Promotes Sleep	ND	2017	28314820	Neural Signalling	Glutamatergic Signalling
Open rectifier K+ channel 1	Promotes Sleep	ND	2017	28682878	Neural Signalling	Potasium Channel
rogdi	Promotes Sleep	Suppresses Rebound Following Deprivation	2017	28900300	Neural Signalling	GABAergic Signalling
Tryptophan hydroxylase neuronal	Promotes Sleep	Promotes Rebound Following Deprivation	2017	28984573	Neural Signalling	Aminergic Signalling
5-hydroxytryptamine (serotonin) receptor 2B	Promotes Sleep	Promotes Rebound Following Deprivation	2017	28984573	Neural Signalling	Aminergic Signalling
F box and leucine-rich-repeat gene 4	Promotes Wakefulness	ND	2017	29174887	Cellular Homoeostasis	Protein Degradation
Jumonji domain containing 5	Promotes Sleep	ND	2018	29339751	Gene Regulation	Chromatin Regulation
Jumonji domain containing 7	Promotes Sleep	ND	2018	29339751	Gene Regulation	Chromatin Regulation
Nucleolar protein 66	Promotes Sleep	ND	2018	29339751	Gene Regulation	Chromatin Regulation
Lysine demethylase 4B	Promotes Wakefulness	ND	2018	29339751	Gene Regulation	Chromatin Regulation
let7	Promotes Sleep	None	2018	29949763	Gene Regulation	MicroRNA
mir-984 stem loop	Promotes Sleep	None	2018	29949763	Gene Regulation	MicroRNA
mir-986 stem loop	Promotes Sleep	None	2018	29949763	Gene Regulation	MicroRNA
mir-977 stem loop	Promotes Sleep	None	2018	29949763	Gene Regulation	MicroRNA
bantam	Promotes Sleep	None	2018	29949763	Gene Regulation	MicroRNA
mir-1003 stem loop	Promotes Sleep	None	2018	29949763	Gene Regulation	MicroRNA
bereft (a.k.a. miR263a)	Promotes Sleep	None	2018	29949763	Gene Regulation	MicroRNA
mir-190 stem loop	Promotes Sleep	Promotes Rebound Following Deprivation	2018	29949763	Gene Regulation	MicroRNA
mir-184 stem loop	Promotes Sleep	None	2018	29949763	Gene Regulation	MicroRNA
mir-955 stem loop	Promotes Sleep	Suppresses Rebound Following Deprivation	2018	29949763	Gene Regulation	MicroRNA
mir-956 stem loop	Promotes Sleep	Suppresses Rebound Following Deprivation	2018	29949763	Gene Regulation	MicroRNA
mir-2b-1 stem loop	Promotes Sleep	None	2018	29949763	Gene Regulation	MicroRNA
mir-981 stem loop	Promotes Sleep	None	2018	29949763	Gene Regulation	MicroRNA
mir-1013 stem loop	Promotes Sleep	None	2018	29949763	Gene Regulation	MicroRNA
mir-992 stem loop	Promotss Sleep	None	2018	29949763	Gene Regulation	MicroRNA
mir-281–2 stem loop	Promotes Sleep	None	2018	29949763	Gene Regulation	MicroRNA
mir-962 stem loop	Promotes Sleep	None	2018	29949763	Gene Regulation	MicroRNA
mir-972 stem loop	Promotes Wakefulness	None	2018	29949763	Gene Regulation	MicroRNA
mir-954 stem loop	Promotes Wakefulness	None	2018	29949763	Gene Regulation	MicroRNA
mir-275 stem loop	Promotes Wakefulness	None	2018	29949763	Gene Regulation	MicroRNA
mir-92a stem loop	Promotes Wakefulness	None	2018	29949763	Gene Regulation	MicroRNA
mir-306 stem loop	Promotes Wakefulness	None	2018	29949763	Gene Regulation	MicroRNA
mir-92b stem loop	Promotes Wakefulness	None	2018	29949763	Gene Regulation	MicroRNA
mir-305 stem loop	Promotes Wakefulness	None	2018	29949763	Gene Regulation	MicroRNA
mir-310 stem loop	Promotes Wakefulness	None	2018	29949763	Gene Regulation	MicroRNA
mir-281–1 stem loop	None	Suppresses Rebound Following Deprivation	2018	29949763	Gene Regulation	MicroRNA
mir-313 stem loop	None	Suppresses Rebound Following Deprivation	2018	29949763	Gene Regulation	MicroRNA
mir-318 stem loop	None	Suppresses Rebound Following Deprivation	2018	29949763	Gene Regulation	MicroRNA
mir-957 stem loop	None	Suppresses Rebound Following Deprivation	2018	29949763	Gene Regulation	MicroRNA
mir-308 stem loop	None	Suppresses Rebound Following Deprivation	2018	29949763	Gene Regulation	MicroRNA
mir-1014 stem loop	None	Suppresses Rebound Following Deprivation	2018	29949763	Gene Regulation	MicroRNA
Innexin 6	Promotes Sleep	ND	2018	30109983	Neural Signalling	Gap Junctions
pudgy	Promotes Sleep	Promotes Rebound Following Deprivation	2018	30186232	Cellular Homoeostasis	Lipid Metabolism
minidiscs	Promotes Wakefulness	ND	2018	30016498	Cellular Homoeostasis	Amino Acid Transport
Juvenile hormone Inducible-21	Promotes Wakefulness	ND	2018	30016498	Cellular Homoeostasis	Amino Acid Transport
eiger	Promotes Sleep	Promotes Rebound Following Deprivation	2018	30379810	Cellular Homoeostasis	Cytokine Signalling
wengen	Promotes Sleep	Promotes Rebound Following Deprivation	2018	30379810	Cellular Homoeostasis	Cytokine Signalling
Phosphoribosylformylglycinamidine synthase (a.k.a. Ade2)	Promotes Sleep	None	2018	30249751	Cellular Homoeostasis	Purine Biosynthesis
Excitatory amino acid transporter 2	Promotes Wakefulness	None	2018	30416062	Cellular Homoeostasis	Amino Acid Transport
Neurocalcin	Promotes Sleep	ND	2019	30865587	Neural Signalling	Calcium Signalling
Serine hydroxymethyl transferase	Promotes Sleep	ND	2019	31064979	Cellular Homoeostasis	Amino Acid Synthesis
Serine racemase	Promotes Sleep	None	2019	31064979	Cellular Homoeostasis	Amino Acid Synthesis
metabotropic GABA-B receptor subtype 3	Promotes Wakefulness	ND	2019	31313987	Neural Signalling	GABAergic Signalling
L-threonine dehydrogenase	Promotes Wakefulness	ND	2019	31313987	Cellular Homoeostasis	Amino Acid Catabolism
noktochor	Promotes Sleep	None	2019	31353186	Not Known	Likely Intercellular Signalling
metabotropic Glutamate Receptor	Promotes Sleep	None	2019	31418019	Neural Signalling	Glutamatergic Signalling
eukaryotic translation initiation factor 2 subunit alphaF-2α kinase	Promotes Sleep	ND	2020	32169212	Cellular Homoeostasis	Kinase Signalling
unpaired 2	Promotes Sleep	ND	2020	32745077	Cellular Homoeostasis	Cytokine Signalling
mir-276a stem loop	Promotes Wakefulness	ND	2021	33337563	Gene Regulation	MicroRNA
stuxnet	Promotes Wakefulness	Suppresses Rebound Following Deprivation	2021	33410264	Cellular Homoeostasis	Protein Degradation
Polycomb	Promotes Sleep	ND	2021	33410264	Gene Regulation	Chromatin Regulation
Octopamine β1 receptor	Promotes Wakefulness	Suppresses Rebound Following Deprivation	2021	33410264	Neural Signalling	Aminergic Signalling
Octopamine β2 receptor	Promotes Wakefulness	Suppresses Rebound Following Deprivation	2021	33410264	Neural Signalling	Aminergic Signalling
Octopamine β3 receptor	Promotes Wakefulness	Suppresses Rebound Following Deprivation	2021	33410264	Neural Signalling	Aminergic Signalling
blue cheese	Promotes Wakefulness	ND	2021	34085929	Cellular Homoeostasis	Vesicle Trafficking
Autophagy-related 1	Promotes Wakefulness	ND	2021	34085929	Cellular Homoeostasis	Autophagy
Heat shock protein 70 cognate 3 (a.k.a., BiP)	Promotes Wakefulness	ND	2021	34085929	Cellular Homoeostasis	Endoplasmic Reticulum Chaperone
Autophagy-related 10	Promotes Wakefulness	ND	2021	34085929	Cellular Homoeostasis	Autophagy
Autophagy-related 8b	Promotes Wakefulness	ND	2021	34085929	Cellular Homoeostasis	Autophagy
Autophagy-related 7	Promotes Wakefulness	ND	2021	34085929	Cellular Homoeostasis	Autophagy
Autophagy-related 12	Promotes Wakefulness	ND	2021	34085929	Cellular Homoeostasis	Autophagy
Another Drosophila Unc-51-like kinase	Promotes Wakefulness	ND	2021	34085929	Cellular Homoeostasis	Autophagy
Atf6	Promotes Wakefulness	ND	2021	34085929	Gene Regulation	Transcription Factor
Dram	Promotes Wakefulness	ND	2021	34085929	Cellular Homoeostasis	Autophagy
wacky	Promotes Wakefulness	ND	2021	34085929	Cellular Homoeostasis	Autophagy
kismet	Promotes Sleep	ND	2021	34088660	Gene Regulation	Chromatin Regulation
cacophony	Promotes Sleep	ND	2021	34015490	Neural Signalling	Calcium Channel
nicotinic Acetylcholine Receptor α2	Promotes Sleep	ND	2021	33493349	Neural Signalling	Cholinergic Signalling
nicotinic Acetylcholine Receptor β2	Promotes Sleep	ND	2021	33493349	Neural Signalling	Cholinergic Signalling
nicotinic Acetylcholine Receptor α1	Promotes Sleep	ND	2021	33493349	Neural Signalling	Cholinergic Signalling
nicotinic Acetylcholine Receptor β1	Promotes Sleep	ND	2021	33493349	Neural Signalling	Cholinergic Signalling
nicotinic Acetylcholine Receptor α5	Promotes Wakefulness	ND	2021	33493349	Neural Signalling	Cholinergic Signalling
uncoordinated 79	Promotes Wakefulness	ND	2021	34849820	Neural Signalling	Sodium Channel Subunit
Methoprene-tolerant	Promotes Wakefulness	ND	2021	34376377	Gene Regulation	Juvenile Hormone Receptor
Mesencephalic astrocyte-derived neurotrophic factor	Promotes Sleep	ND	2021	33666288	Cellular Homoeostasis	Neurotrophic Factor
14-3-3ε	Promotes Sleep	ND	2021	34575915	Cellular Homoeostasis	Second Messenger Signalling
Hugin	Promotes Sleep	Suppresses Rebound Following Deprivation	2021	34782479	Neural Signalling	Neuropeptide Signalling
rumpel	Promotes Wakefulness	ND	2021	34897385	Cellular Homoeostasis	Solute/Sodium Symporter
D-amino acid oxidase 1	Promotes Wakefulness	ND	2021	34922200	Cellular Homoeostasis	Amino Acid Catabolism
GABA transporter	Promotes Wakefulness	Promotes Rebound Following Deprivation	2022	35303417	Neural Signalling	GABAergic Signalling
Lipophorin receptor 1	Promotes Wakefulness	ND	2022	36071487	Cellular Homoeostasis	Lipid Uptake
Lipophorin receptor 2	Promotes Wakefulness	ND	2022	36071487	Cellular Homoeostasis	Lipid Uptake
Dab adaptor protein (a.k.a.disabled)	Promotes Wakefulness	ND	2022	36071487	Cellular Homoeostasis	Tyrosine Kinase Signalling
Serotonin transporter	Promotes Wakefulness	ND	2022	36409783	Neural Signalling	Aminergic Signalling
U snoRNA host gene 4	Promotes Wakefulness	ND	2022	36451091	Gene Regulation	Noncoding RNA
Desaturase 1	Promotes Wakefulness	ND	2022	35199930	Pheromone Signalling	Cuticular Hydrocarbon Synthesis
Cytochrome P450 4g1	Promotes Wakefulness	ND	2022	35199930	Pheromone Signalling	Cuticular Hydrocarbon Synthesis
pickpocket 23	Promotes Wakefulness	ND	2022	35199930	Pheromone Signalling	Pheromone Perception
pickpocket 29	Promotes Wakefulness	ND	2022	35199930	Pheromone Signalling	Pheromone Perception
Connectin	Promotes Sleep	ND	2023	36608130	Cellular Homoeostasis	Cell Adhesion, Synaptic Development
daughterless	Promotes Sleep	ND	2023	36608130	Gene Regulation	Transcription Factor
homothorax	Promotes Sleep	ND	2023	36608130	Gene Regulation	Transcription Factor
G protein beta subunit 13F	Promotes Wakefulness	ND	2023	36608130	Cellular Homoeostasis	G Protein Signalling
twister	Promotes Wakefulness	ND	2023	36608130	Gene Regulation	Alternative Splicing
Phosphatidylinositol glycan anchor biosynthesis class Q	Promotes Wakefulness	ND	2023	36608130	Cellular Homoeostasis	Membrane Anchor
Phosphatidylinositol glycan anchor biosynthesis class Z	Promotes Wakefulness	None	2023	36608130	Cellular Homoeostasis	Membrane Anchor
Phosphatidylinositol glycan anchor biosynthesis class O	Promotes Wakefulness	ND	2023	36608130	Cellular Homoeostasis	Membrane Anchor
Phosphatidylinositol glycan anchor biosynthesis class C	Promotes Wakefulness	ND	2023	36608130	Cellular Homoeostasis	Membrane Anchor
Phosphatidylinositol glycan anchor biosynthesis class G	Promotes Wakefulness	ND	2023	36608130	Cellular Homoeostasis	Membrane Anchor
Phosphatidylinositol glycan anchor biosynthesis class M	Promotes Wakefulness	ND	2023	36608130	Cellular Homoeostasis	Membrane Anchor
Ecdysone receptor	Promotes Sleep	ND	2023	36719183	Gene Regulation	Nuclear Hormone Receptor
Ecdysone-induced protein 75B	Promotes Sleep	ND	2023	36719183	Gene Regulation	Nuclear Hormone Receptor
ftz transcription factor 1	Promotes Sleep	ND	2023	36719183	Gene Regulation	Likely Nuclear Hormone Receptor
Hormone receptor 3	Promotes Sleep	ND	2023	36719183	Gene Regulation	Nuclear Hormone Receptor
retinal degeneration B	Promotes Sleep	ND	2023	36586155	Cellular Homoeostasis	G Protein Signalling
Cytoplasmic FMR1 interacting protein	Promotes Sleep	Promotes Rebound Following Deprivation	2023	36808152	Cellular Homoeostasis	Cytoskeleton Regulation
allnighter	Promotes Sleep	ND	2023	37217484	Cellular Homoeostasis	Pseudokinase
ebony	Promotes Wakefulness	ND	2023	37369755	Neural Signalling	Aminergic Signalling, Cuticle Formation
Pallidin	Promotes Sleep	ND	2023	37682712	Cellular Homoeostasis	Lysosome Biogenesis
Biogenesis of lysosome-related organelles complex 1, subunit 1	Promotes Sleep	ND	2023	37682712	Cellular Homoeostasis	Lysosome Biogenesis
Biogenesis of lysosome-related organelles complex 1, subunit 2	Promotes Sleep	ND	2023	37682712	Cellular Homoeostasis	Lysosome Biogenesis
Dysbindin	Promotes Sleep	ND	2023	37682712	Cellular Homoeostasis	Lysosome Biogenesis
Juvenile hormone Inducible-21	Promotes Sleep	ND	2023	37682712	Cellular Homoeostasis	Amino Acid Transport
minidiscs	Promotes Sleep	ND	2023	37682712	Cellular Homoeostasis	Amino Acid Transport
raptor	Promotes Sleep	ND	2023	37682712	Cellular Homoeostasis	TOR Signalling
mechanistic Target of rapamycin	Promotes Sleep	ND	2023	37682712	Cellular Homoeostasis	TOR Signalling
moody	Promotes Sleep	ND	2023	37831742	Cellular Homoeostasis	Blood Brain Barrier
G protein alpha subunit o	Promotes Sleep	ND	2023	37831742	Cellular Homoeostasis	G Protein Signalling
locomotion defects	Promotes Sleep	ND	2023	37831742	Cellular Homoeostasis	G Protein Signalling
Lachesin	Promotes Sleep	ND	2023	37831742	Cellular Homoeostasis	Septate Junctions
Neuroglian	Promotes Sleep	ND	2023	37831742	Cellular Homoeostasis	Cell Adhesion, Synaptic Development
Protein kinase, cAMP-dependent, catalytic subunit 1	Promotes Sleep	ND	2023	37831742	Cellular Homoeostasis	Kinase Signalling

**Table 3.** Strong candidates for genes governing homeostatic sleep regulation. This table lists the genes from Tables 1 and 2 that produce the congruent effects on baseline sleep and sleep rebound following deprivation predicted by the two-process model of sleep regulation.

Gene Name	Role in Baseline Sleep	Role in Sleep Rebound	Year	PMID	General Mechanism	Specific Mechanism
Tryptophan hydroxylase neuronal	Promotes Sleep	Promotes Rebound Following Deprivation	2017	28984573	Neural Signalling	Aminergic Signalling
Octopamine β3 receptor	Promotes Wakefulness	Suppresses Rebound Following Deprivation	2021	33410264	Neural Signalling	Aminergic Signalling
Octopamine β2 receptor	Promotes Wakefulness	Suppresses Rebound Following Deprivation	2021	33410264	Neural Signalling	Aminergic Signalling
Octopamine β1 receptor	Promotes Wakefulness	Suppresses Rebound Following Deprivation	2021	33410264	Neural Signalling	Aminergic Signalling
fumin	Promotes Sleep	Promotes Rebound Following Deprivation	2005, 2008	16093388, 18457233	Neural Signalling	Aminergic Signalling
5-hydroxytryptamine (serotonin) receptor 2B	Promotes Sleep	Promotes Rebound Following Deprivation	2017	28984573	Neural Signalling	Aminergic Signalling
cAMP Response Element Binding Protein (CREB)	Promotes Wakefulness	Suppresses Rebound Following Deprivation	2001	11687816	Cellular Homoeostasis	cAMP Signalling
cyclin A	Promotes Sleep	Promotes Rebound Following Deprivation	2012	22461610	Cellular Homoeostasis	Cell Cycle Control
wengen	Promotes Sleep	Promotes Rebound Following Deprivation	2018	30379810	Cellular Homoeostasis	Cytokine Signalling
eiger	Promotes Sleep	Promotes Rebound Following Deprivation	2018	30379810	Cellular Homoeostasis	Cytokine Signalling
Cytoplasmic FMR1 interacting protein	Promotes Sleep	Promotes Rebound Following Deprivation	2023	36808152	Cellular Homoeostasis	Cytoskeleton Regulation
rhomboid	Promotes Sleep	Promotes Rebound Following Deprivation	2007	17694052	Cellular Homoeostasis	Growth Factor Signalling
pudgy	Promotes Sleep	Promotes Rebound Following Deprivation	2018	30186232	Cellular Homoeostasis	Lipid Metabolism
mir-190 stem loop	Promotes Sleep	Promotes Rebound Following Deprivation	2018	29949763	Gene Regulation	MicroRNA
Sex peptide receptor	Promotes Sleep	Promotes Rebound Following Deprivation	2014	25333796	Neural Signalling	Neuropeptide Signalling
Neurexin 1	Promotes Sleep	Promotes Rebound Following Deprivation	2016	27905548	Neural Signalling	Neuropeptide Signalling
Myoinhibiting peptide precursor	Promotes Sleep	Promotes Rebound Following Deprivation	2014	25333796	Neural Signalling	Neuropeptide Signalling
yellow-achaete intergenic RNA	Promotes Sleep	Promotes Rebound Following Deprivation	2011	21775470	Gene Regulation	Noncoding RNA
Ecdysone receptor	Promotes Sleep	Promotes Rebound Following Deprivation	2010	20215472	Gene Regulation	Nuclear Hormone Receptor, Steroid Signalling
sleepless (a.k.a., quiver)	Promotes Sleep	Promotes Rebound Following Deprivation	2008	18635795	Neural Signalling	Potassium Channel
stuxnet	Promotes Wakefulness	Suppresses Rebound Following Deprivation	2021	33410264	Cellular Homoeostasis	Protein Degradation
insomniac	Promotes Sleep	Promotes Rebound Following Deprivation	2012	23055946	Cellular Homoeostasis	Protein Degradation
Cullin-3	Promotes Sleep	Promotes Rebound Following Deprivation	2011, 2012	22196332, 23055946	Cellular Homoeostasis	Protein Degradation

To simplify the summary of this work, sleep-regulating genes have been classified as promoting sleep if the mutation (null or hypomorphic) or knockdown decreases sleep. I have omitted some categories of genes to focus on the most promising candidate mediators of sleep homoeostasis. Circadian clock genes have been omitted from this list, as the circadian clock is a primary sleep regulator and is, therefore, expected to produce strong sleep phenotypes independently of homoeostatic control (see below). Only genes that affect baseline sleep or rebound following deprivation are included in Table 2. Mutations that affect sleep bout duration, number, or latency have been omitted if they do not alter total sleep. Several genes reported in the sleep literature that could not be identified or disambiguated on flybase.org have not been included. Genetic manipulations affecting sleep only within specific contexts (e.g. under starvation or genetic disease model backgrounds) are not included here. Genes implicated only by over-expression, or the expression of constitutively active mutant gene forms, have also been omitted from the tables. Gene manipulations that produced sleep phenotypes only when other genes were simultaneously manipulated are also not included. Genes implicated based only on the excitation or inhibition of the neurons that express it are also omitted from the table. Genes whose loss of function or knockdown resulted in changes in daytime or night-time sleep without affecting total sleep have been omitted from the table. Genes whose knockdown produces opposing sleep phenotypes in different cell types, such as the amino acid transporter *Juvenile hormone Inducible-21* [49] or the GABA-B-R2 receptor [50,51], are not included in Table 2. When mutated or knocked down, genes that have displayed opposing sleep phenotypes in different studies are also not included in Table 2. Finally, receptor-encoding genes implicated only through the feeding of receptor agonists or antagonists are not included in Table 2.

## A large and growing list of Drosophila ‘Sleep genes’

In 2015, Afonso and colleagues provided a comprehensive review of sleep-regulating genes in Drosophila, identifying 73 genes [52]. As of February 2024, the fly sleep field had identified over two hundred genes whose mutation or knockdown affects the daily amount of sleep or the amount of sleep rebound observed following deprivation (Tables 1 and 2 and Supplemental Table S1). I have categorized these genes into three general and somewhat arbitrary categories under the ‘General Mechanism’ heading in the tables. The first category consists of genes whose products contribute to cellular homoeostasis. This category includes gene products related to basic cellular processes, such as biosynthesis (not including the synthesis of neurotransmitters), cellular trafficking, metabolism, second messenger signalling, cell growth, cell division, structural proteins mediating tissue integrity, and others. The second category consists of genes encoding components supporting neural signalling. This category includes gene products that regulate membrane voltage, action potential firing, and synaptic function, including ion channels, enzymes necessary for synthesizing neurotransmitters and neuromodulators, neuropeptides, receptors, and others. The third category consists of genes whose primary function is to regulate the expression of other genes. Included are transcription factors, nuclear hormone receptors, micro RNAs, gene products governing chromatin regulation, gene splicing, etc.

Mutations in the nine genes identified by unbiased genetic screens all produced a decrease in total baseline sleep, suggesting that they usually act to promote sleep (Table 1). In only three instances, the genes *fumin* [33], *sleepless* (a.k.a. *quiver*) [31], and *insomniac* [53], did these mutations result in a change in sleep rebound following deprivation, reducing sleep rebound. For *Shaker* [29] and *Hyperkineti*c [30], no effects on sleep rebound were observed following deprivation. To my knowledge, rebound has not been assessed for the remaining five genes identified in forward genetic screens. The products of ‘sleep genes’ identified in forward genetic screens govern either neural signalling (six genes) or cellular homoeostasis (three genes), with none involved directly in regulating gene expression.

For the 197 genes identified by reverse genetic methods, the knockdown or disruption of 110 genes decreased baseline sleep, and the knockdown or disruption of 74 genes increased baseline sleep. For the remaining 12 genes, knockdown or disruption caused no change in baseline sleep but did produce decreases (9 genes) or increases (3 genes) in rebound following sleep deprivation (Table 2). The nearly 200 genes identified by reverse genetic methods encode gene products involved in all three general mechanisms, with 75 mediating cellular homoeostasis, 59 gene regulation, 58 mediating neural signalling, and five additional genes that could not be easily placed in these categories.

**Figure 2.**
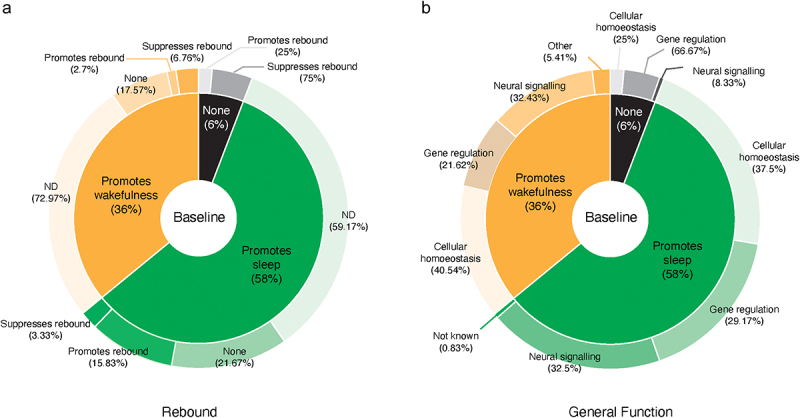
(a) a pie-donut chart summarizing the complete list of ‘Sleep genes’ and their relation to baseline sleep amount and rebound following deprivation. The centre of the chart indicates the breakdown of sleep-promoting genes (dark green), wake-promoting genes (dark orange), and genes that produced no effect on baseline sleep (black). The outer edge of the chart indicates the breakdown of sleep rebound effects for each of the three central baseline sleep categories. For the outer chart, ‘ND’ indicates no deprivation experiments have been reported, and ‘None’ indicates that such experiments revealed no significant effect on sleep rebound. (b)a pie-donut chart summarizing the complete list of ‘sleep genes’ and their relation to baseline sleep amount and rebound following deprivation. The centre of the chart indicates the breakdown of sleep-promoting genes (dark green), wake-promoting genes (dark orange), and genes that produced no effect on baseline sleep (black). The outer edge of the chart indicates the breakdown of General mechanisms for each of the three central baseline sleep categories. For the outer chart, ‘Other’ indicates functions that could not be easily assigned to the three main categories used in the tables. One sleep-promoting gene is of unknown function and is indicated as ‘Not known’ in the outer edge.

An examination of the combined list of sleep-regulating genes (Figure 2a; Supplemental Table S1) reveals that 120 promote sleep, 74 promote wakefulness, and 12 are not required for normal baseline sleep/wake levels. Thus, approximately two-thirds of the genes identified thus far promote sleep under baseline conditions, and approximately one-third promote wakefulness. No assessment of rebound following sleep deprivation has been reported for most identified sleep-regulating genes (126 genes). Of the 80 genes for which rebound experiments have been reported, nearly half (39 genes) displayed no rebound phenotype, 23 genes were found to promote rebound, and 18 to suppress it. A visual summary of this analysis is shown in Figure 2.

The list of fly’s sleep-regulating genes includes many genes whose homologs influence sleep in mammals. As previously reviewed by Zimmerman and colleagues, genes involved in growth factor signalling (e.g. *rhomboid*), second messenger signalling (e.g. *CrebB*), and aminergic signalling (e.g. *Dat*, a.k.a. *speck* or *AANAT1*) play conserved roles in fly and rodent sleep regulation and gene products related to the unfolded protein response display increased expression following sleep deprivation (e.g. *Heat shock protein 70 cognate 2*, a.k.a., *Bip*) [54]. Furthermore, genes regulating protein ubiquitination, such as *insomniac* and *Cullin-3*, regulate sleep in both flies and mammals [55]. The conserved nature of genetic sleep regulation is further illustrated by studies identifying sleep-regulating genes by examining fly homologs of genes implicated in human GWAS studies. For example, Eiman et al. (2024) identified genes regulating sleep latency using this approach [56].

## Comparing genetic screens for circadian timekeeping and sleep regulation

Sleep is primarily governed by two regulatory mechanisms: a sleep homeostat and a circadian clock. The circadian clock genes discovered in *Drosophila* are highly conserved [57], and homologs of these genes govern circadian timekeeping in humans [58]. Homoeostatic regulation of sleep is likewise thought to be evolutionarily ancient and conserved [59], and there is every reason to believe that the identification of genes governing such sleep regulation in the fly will lead to the identification of conserved genetic mechanisms. The behavioural methods used to measure sleep and circadian timekeeping are based on the same simple behavioural metric: infrared beam crossing data from isolated flies [27]. Despite using the same behavioural and genetic methods, the outcomes of genetic screens for circadian and homoeostatic sleep regulation have been very different.

One clear difference between clock and sleep screens is the number of genes identified, with only approximately a dozen genes implicated in circadian timekeeping compared to ~ 200 sleep-regulating genes. A second significant difference is the primary genetic approach used to identify sleep and clock genes. Nearly all the clock genes discovered in *Drosophila* were identified based on unbiased forward genetic screens [24]. In contrast, only nine of approximately 200 sleep-regulating genes were identified by forward genetics (Table 1), with the remaining identified based on reverse genetic methods, typically RNAi-mediated knockdown of gene expression (Table 2). Thus, the majority of the genes implicated in sleep regulation were identified through the testing of candidate genes, chosen based on their already established molecular functions. Much of the reverse genetic screening was also done in cell types already implicated in sleep regulation. Consequently, in contrast to circadian genes, most ‘sleep genes’ were not identified in unbiased screens but by examining candidate genes chosen based on *a priori* assumptions regarding the genes and cell types likely to mediate sleep regulation. The sleep and circadian fields’ differential reliance on reverse genetics is likely a matter of historical timing. The establishment of fly sleep, decades after circadian rhythms were first examined, coincided with the rapid and sustained development of reverse genetic tools that offered an attractive alternative to the much more labour-intensive forward genetic approach.

A third significant difference is in clock and ‘sleep gene’ cellular/molecular functions. The clock genes discovered in forward genetic screens were subsequently found to cooperate within the selfsame cellular process: rhythmic gene expression through transcriptional-translational feedback loops [57]. This is reflected by the fact that the overwhelming majority of clock genes function via the regulation of gene expression. In contrast, genes implicated in sleep regulation play many distinct biological roles with no apparent convergence on a single molecular/cellular mechanism (Tables 1 and 2; Figure 2b). Identifying the cellular and molecular mechanisms underlying homoeostatic sleep regulation remains a central goal of sleep science.

One recent and highly compelling model [60,61] has incorporated the three general biological mechanisms used to categorize ‘sleep genes’ in Tables 1 and 2. According to this model, metabolic changes within neurons driven by aminergic inputs signalling wakefulness are coupled with changes in potassium channels that alter the firing of sleep-promoting neurons of the central brain [60,61]. However, independent studies have recently called this model into serious question [62,63]. Thus, despite 25 years of genetic studies and the identification of over 200 sleep-regulating genes, the field still lacks a widely accepted molecular/cellular model of fly sleep homoeostasis. The most fundamental difference between the genetics of circadian timekeeping and sleep regulation, therefore, is success. Genetic screens for clock mutants have revealed a widely accepted molecular/cellular model of circadian timekeeping. In contrast, the identification of ~ 200 sleep-regulating genes has not yet led to a widely accepted model of sleep homoeostasis. Why have screens for homoeostatic sleep and circadian clock genes had such distinct outcomes? The relatively large number of ‘sleep genes’ identified by the field may reflect that sleep regulation is distributed across many molecular processes and cell types. Thus, unlike circadian timekeeping, homoeostatic sleep regulation may depend critically on many genes regulating diverse and redundant molecular/cellular processes. However, the failure of ‘sleep genes’ to converge on accepted cellular/molecular mechanisms may indicate that we have not yet identified *bona fide* regulators of sleep homoeostasis.

A salient difference between screens for sleep and circadian mutants is the environmental condition under which circadian rhythms and sleep have been observed. The core phenomenon of circadian timekeeping is assayed under constant darkness and temperature so that the circadian clock’s endogenous speed can be measured without environmental time cues or influences [27,64]. Under such conditions, there are few processes outside of the core circadian clock mechanism that would change the strength or speed of the circadian clock. In contrast, sleep has been studied almost exclusively under light/dark cycles consisting of 12 hours of bright fluorescent light followed by 12 hours of complete darkness. The stark transitions associated with such cycles significantly affect activity and rest [12]. This strong light-induced modulation of sleep likely masks homoeostatic processes, making it challenging to detect homoeostatic mutants in genetic screens. Furthermore, the intensity of environmental light strongly influences daytime sleep amount [65]. However, the light intensity employed by investigators is not typically reported, which makes it challenging to eliminate light’s influence as a reason for a given gene’s contribution to total sleep amount.

Differences in how sleep and circadian rhythms are measured are also likely to explain, to some extent, the distinct outcomes of sleep and circadian screens. For circadian timekeeping under constant conditions, screens have employed time-series analyses that quantify the endogenous rhythms’ strength and periodicity (i.e., speed). These metrics are not strongly dependent on the number of beam crossings made by any fly strain, meaning that mutations that caused increases or decreases in daily activity without eliminating timekeeping or altering clock speed would not pass the selection criteria for identifying clock mutants. This afforded clock screens a powerful specificity for *bona fide* changes in circadian timekeeping. In contrast, sleep screens have relied on the amount of time spent asleep, defined as the time spent in bouts of inactivity lasting five minutes or longer. Thus, any mutation or genetic manipulation that significantly increases or decreases total sleep time will implicate the gene in question as a sleep-regulating gene, regardless of whether it mediates sleep homoeostasis or acts via other sleep-modulating processes, of which there appear to be many [66]. This difference likely explains, to a significant degree, why we should expect to find a significantly larger number of genes governing sleep regulation than circadian timekeeping. For reasons described above, creens for clock mutants were sensitized to reveal changes in one fundamental process (endogenous timekeeping), while those for sleep reflect changes in myriad sleep-regulating and sleep-modulating pathways. In addition, the genetic background significantly affects sleep homoeostasis [67]. Thus, some ‘sleep genes’ may only manifest their impact on specific mutant backgrounds.

Closely related to this point is the high likelihood that the different outcomes of sleep and circadian screens reflect fundamental differences in the two processes. To be useful, clocks need to be buffered from external factors and should not weaken, speed up, or slow down in response to frequently experienced external or internal influences [68]. Such endogenous timekeeping persists even in the face of significant changes in external conditions or internal states, e.g. across a wide range of temperatures or underfed or starved conditions. In contrast, sleep is notorious for its sensitivity to external and internal influences. In human subjects, changes in internal states such as psychological state, discomfort/pain, illness, hormonal changes, and external states such as noise, light, temperature, and social interactions can all influence sleep [69]. The same applies to fly sleep [66]. Many physiological processes are expected to modulate sleep without playing a significant role in its homoeostatic regulation, and genetic manipulations in these processes are expected to produce changes in the amount of daily sleep. Therefore, many and possibly all of the ‘sleep genes’ listed in Tables 1 and 2 may contribute to regulatory pathways that indirectly influence sleep without contributing directly to sleep homoeostasis.

In summary, the relatively large number of ‘sleep genes’ identified over the last 25 years is likely explained by differences in screening methodologies and the fact that daily sleep amount is influenced by myriad molecular/cellular processes, of which homoeostatic control is only one. Future progress in uncovering the mechanistic basis of sleep homoeostasis will depend critically on using methods that sensitize screens for homoeostatic regulators. Given the established power of such screens in *Drosophila*, developing screening methods that differentiate changes in homoeostatic processes from changes in modulatory processes should, therefore, be a central goal of future work on the genetics of *Drosophila* sleep.

## Which of the 206 identified ‘Sleep genes’ are the strongest candidates as mediators of sleep homoeostasis?

Though many, if not all, of the genes listed in Tables 1 and 2 may ultimately prove unrelated to homoeostatic sleep regulation, some may represent *bona fide* sleep homoeostasis genes. The two-process model of sleep regulation, first proposed by Borbély, accounts remarkably well for human sleep under both baseline and deprivation conditions. A central feature of this model is that the same process (process S) mediates both normal daily increases in sleep pressure and the rebound sleep observed following sleep deprivation [3,70]. Based on this model, we would expect genes involved in sleep homoeostasis to produce changes in both baseline sleep and rebound. Furthermore, based on a straightforward interpretation of this model, we would expect congruent effects on baseline and rebound sleep. That is, genes that promote sleep would also promote rebound following deprivation, and genes that promote wakefulness would suppress rebound.

Of the 68 genes that produced baseline sleep phenotypes also tested for rebound phenotypes, only 29 displayed rebound phenotypes (Tables 1 and 2 and references therein). Six displayed incongruent effects on baseline sleep and rebound, with four sleep-promoting genes suppressing rebound and two wake-promoting genes promoting rebound. It has been suggested that the failure to see consistent effects of genes on both baseline sleep and rebound indicates that distinct processes, and therefore genes, govern baseline sleep levels and homoeostatic rebound (e.g [39]). If true, this would mean that the two-process model does not hold for *Drosophila*. However, our recent work [71] suggests that such a model captures the daily amount and timing of fly sleep well and makes remarkably accurate predictions about the effects of altered circadian clocks on sleep. Until a compelling and widely accepted model of sleep homoeostasis emerges, the relationships between the processes governing baseline sleep and sleep rebound following deprivation will remain uncertain.

Despite this uncertainty, the two-process model can be employed to interpret previous work and design new approaches to understanding homoeostatic sleep regulation. Such an approach would reveal if the model holds for flies. Taking the model as a framework for prioritizing the 206 genes listed in Tables 1 and 2, I consider the strongest gene candidates for homoeostatic sleep regulation to be those that produce effects on both baseline sleep and sleep rebound when disrupted and do so with the expected congruence described above. Of the 206 ‘sleep genes’ listed in Tables 1 and 2, only 23 genes produce the predicted congruent effects on baseline sleep and homoeostatic rebound when manipulated or mutated, with 18 representing sleep-promoting and five representing wake-promoting genes. An examination of the gene products encoded by these genes reveals that three of these candidates encode products related to gene regulation, with the remaining 20 split evenly between genes governing cellular homoeostasis and neural signalling (Table 3). Throughout the tables, I have also categorized each gene under the ‘specific mechanism’ heading. Examining the prioritized genes in Table 3 reveals that genes governing aminergic signalling represent the largest group in these strong candidates for sleep homoeostasis genes. Mechanisms of neuropeptide signalling and protein degradation are also well represented by these strong candidates.

Homoeostatic control of sleep persists without circadian timekeeping [7,72], and circadian timekeeping persists without sleep. For this reason, the two-process model assumes that homoeostatic and circadian regulation act independently. However, growing evidence shows that the two processes likely influence each other [73]. For example, loss of function mutations in circadian clock genes result in changes in homoeostatic sleep rebound following deprivation [74], and similar genetic interactions likely exist between the circadian clock and sleep homoeostasis in rodents [75]. Furthermore, a two-process model of fly sleep suggests that genetic changes that alter circadian clock speed may alter homoeostatic sleep processes [71]. Nevertheless, given that these two processes persist without one another, they are expected to be mediated by distinct genetic mechanisms. It is for this reason that I have not included circadian clock genes in the tables here.

## Challenges, recent developments, and future screens for genetic regulators of sleep homeostasis

Several features of fly sleep pose significant challenges to discovering the mechanisms underlying homoeostatic sleep regulation. In mammals, sleep consists of discrete stages with distinct relationships to homoeostatic control [76–78]. Despite growing evidence for a distinct deep sleep stage in the fly [79,80], the field has continued treating sleep as a unitary state of any inactivity lasting 5 minutes or longer. Recent work in my lab suggests that sleep homoeostasis more potently regulates longer, deeper bouts of sleep in flies and that including shorter bouts of sleep in the analysis of sleep amount can obscure the homoeostatic regulation of sleep [81]. Thus, future progress in identifying genes governing homoeostatic sleep regulation will likely require investigators to examine specific sleep stages when using sleep amount and rebound as screening metrics.

Furthermore, as first recognized by Cirelli, methods used to investigate homoeostatic rebound following sleep deprivation produce relatively small magnitude rebounds within a narrow time window [26]. This modest sleep rebound presents a small dynamic range to examine potential changes in homoeostatic control. This modest homoeostatic response in flies is likely due, in large part, to the methods used to prevent sleep in deprivation experiments, which are confounded by the need to provide frequent and intense mechanical or neural stimulation for extended periods [81]. Many of the differences observed between deprived and control flies are expected to be direct effects of the frequent and intense mechanical/neuronal stimulation used to prevent sleep rather than effects of increased sleep pressure in deprived flies. Progress in identifying genetic regulators of sleep homoeostasis will likely require investigators to control for these effects to ensure that the mutants identified in our screens have *bona fide* sleep homoeostasis phenotypes.

Given that many biological processes are expected to modulate sleep amount, using total daily sleep as the primary or sole screening metric is likely to reveal genes not directly involved in sleep homoeostasis that take part in other pathways. Effective screens for sleep homoeostasis mutants will need to incorporate means of differentiating mutations mediating sleep homoeostasis from mutations that modify sleep through different processes. These efforts may benefit from predictions made by a two-process model of fly sleep [71]. New technologies for the observation of sleep and sleep-related behaviours, such as the Ethoscope [82], FlyBox [83], and FlyVISTA [84], offer increased accuracy in sleep measurements and the ability to observe sleep-related behavioural or postural changes, which are not observable using the beam crossing recordings typically used in the field. Furthermore, methods for measuring arousal threshold are now available to the fly sleep researcher [83,85], as are approaches to controlling for sleep-independent effects of mechanical sleep deprivation [81]. Finally, computational approaches now exist to examine sleep stages in the fly [80]. These new technologies will be indispensable for confirming that a given gene contributes significantly to sleep homoeostasis. However, the traditional beam-crossing approach will likely remain the tool of choice for high throughput screening. Though the fly has much more to tell us about the genetic basis of sleep regulation, continued progress in the field will require a critical reassessment of current methods and the adoption of new approaches to validate gene candidates. Such a reassessment will be required if the fly’s enduring promise for discovering conserved mechanisms underlying sleep homoeostasis is to be fully realized. The 25^th^ anniversary of fly sleep seems as good a time as any for such a reassessment.

## Supplementary Material

Supplemental Material

Table S1.xlsx

## Data Availability

N/A. There are no data associated with this review.
