# Supplementary material for: 25 years of Drosophila “Sleep genes”
Source: Fly (Austin). 2025 May 6;19(1):2502180. doi: 10.1080/19336934.2025.2502180 (PMC12064057; doi:10.1080/19336934.2025.2502180)
Supplement: Supplemental Material [file KFLY_A_2502180_SM8841.docx]

**Supplemental Table Legend:**

**Supplemental Table 1: All “Sleep Genes” Included in this Review.** All the genes from Tables 1 and 2 combined, with an additional indication of the genetic methods used for each gene. This combined table is presented as an Excel spreadsheet for easy sorting and examining genes based on the categories listed.

**Table 1:**

| Gene Name | Role in Baseline Sleep | Role in Sleep Rebound | Year | PMID | General Mechanism | Specific Mechanism |
| --- | --- | --- | --- | --- | --- | --- |
| Shaker | Promotes Sleep | None | 2005, 2011 | 15858564, 22196332 | Neural Signalling | Potassium Channel |
| fumin | Promotes Sleep | Promotes Rebound Following Deprivation | 2005, 2008 | 16093388, 18457233 | Neural Signalling | Aminergic Signaling |
| Hyperkinetic | Promotes Sleep | None | 2007 | 17507560 | Neural Signalling | Potassium Channel |
| sleepless (a.k.a., quiver) | Promotes Sleep | Promotes Rebound Following Deprivation | 2008 | 18635795 | Neural Signalling | Potassium Channel |
| insomniac | Promotes Sleep | Promotes Rebound Following Deprivation | 2011 | 22196332, 23055946 | Cellular Homeostasis | Protein Degradation |
| Redeye | Promotes Sleep | ND | 2014 | 24497543 | Neural Signalling | Cholinergic Signaling |
| wide awake | Promotes Sleep | ND | 2014 | 24631345 | Neural Signalling | GABAergic Signaling |
| taranis | Promotes Sleep | ND | 2015 | 26096977 | Cellular Homeostasis | Cell Cycle Regulation |
| argus | Promotes Sleep | ND | 2021 | 34085929 | Cellular Homeostasis | Autophagy |

**Table 2:**

| Gene | Role in Baseline Sleep | Role in Sleep Rebound | Year | PMID | General Mechanism | Specific Mechanism |
| --- | --- | --- | --- | --- | --- | --- |
| speck (a.k.a. Dat and AANAT1) | None | Suppresses Rebound Following Deprivation | 2000, 2020 | 10710313, 32955431 | Neural Signalling | Aminergic Signaling |
| cAMP Response Element Binding Protein B (CrebB) | Promotes Wakefulness | Suppresses Rebound Following Deprivation | 2001 | 11687816 | Second Messenger Signalling | cAMP Signaling |
| dunce | Promotes Sleep | ND | 2001 | 11687816 | Second Messenger Signalling | cAMP Signaling |
| rutabaga | Promotes Wakefulness | ND | 2001 | 11687816 | Second Messenger Signalling | cAMP Signaling |
| 5-hydroxytryptamine (serotonin) receptor 1A | Promotes Sleep | None | 2006, 2017 | 16753559, 28984573 | Neural Signalling | Aminergic Signaling |
| Relish | Promotes Sleep | None | 2007 | 17520783 | Gene Regulation | Inflammation/Immunity |
| Heat shock protein 70 cognate 3 (a.k.a., BiP and Grp78) | None | Promotes Rebound Following Deprivation | 2007 | 17552370 | Cellular Homeostasis | Unfolded Protein Response |
| rhomboid | Promotes Sleep | Promotes Rebound Following Deprivation | 2007 | 17694052 | Cellular Homeostasis | Growth Factor Signaling |
| Resistant to dieldrin | Promotes Sleep | ND | 2008 | 18223647, 19038223 | Neural Signalling | GABAergic Signaling |
| Tyrosine decarboxylase 2 | Promotes Wakefulness | ND | 2008 | 18799671 | Neural Signalling | Aminergic Signaling |
| Tyramine β hydroxylase | Promotes Wakefulness | ND | 2008 | 18799671 | Neural Signalling | Aminergic Signaling |
| Activating transcription factor-2 | Promotes Sleep | ND | 2008 | 18694958 | Gene Regulation | Transcription Factor, Stress Response |
| Pigment dispersing factor | Promotes Wakefulness | ND | 2008 | 19038223 | Neural Signalling | Neuropeptide Signaling |
| Fragile X messenger ribonucleoprotein 1 | Promotes Wakefulness | Promotes Rebound Following Deprivation | 2009 | 19228950 | Gene Regulation | RNA Translation and Trafficking, Neural Development and Plasticity |
| Dopamine 1-like receptor 1 | Promotes Wakefulness | ND | 2009 | 19945394 | Neural Signalling | Aminergic Signaling |
| Ecdysone receptor | Promotes Sleep | Promotes Rebound Following Deprivation | 2010 | 20215472 | Gene Regulation | Nuclear Hormone Receptor, Steroid Signaling |
| brummer | None | Suppresses Rebound Following Deprivation | 2010 | 20824166 | Cellular Homeostasis | Lipid metabolism and Storage |
| Lipid storage droplet-2 | None | Promotes Rebound Following Deprivation | 2010 | 20824166 | Cellular Homeostasis | Lipid metabolism and Storage |
| Elongator complex protein 3 | Promotes Sleep | ND | 2010 | 20626565 | Gene Regulation | Chromatin Regulation |
| Angiotensin-converting enzyme-related | Promotes Sleep | ND | 2011 | 21270318 | Cellular Homeostasis | Peptidase, Heart Rate Regulation, Aging |
| G protein alpha subunit o | Promotes Sleep | ND | 2011 | 21358844 | Cellular Homeostasis | G Protein Signaling |
| bunched | None | Promotes Rebound Following Deprivation | 2011 | 21549599 | Gene Regulation | Transcription Factor |
| Notch | None | Suppresses Rebound Following Deprivation | 2011 | 21549599 | Gene Regulation | Cell Fate Regulation |
| sarah | Promotes Sleep | ND | 2011 | 21900555 | Cellular Homeostasis | Calcium Signaling, Mieosis, Courtship Behavior |
| Calcineurin A at 14F | Promotes Sleep | None | 2011 | 21900555, 21917797 | Cellular Homeostasis | Calcium Signaling |
| Calcineurin B | Promotes Sleep | None | 2011 | 21900555, 21917797 | Cellular Homeostasis | Calcium Signaling |
| yellow-achaete intergenic RNA | Promotes Sleep | Promotes Rebound Following Deprivation | 2011 | 21775470 | Gene Regulation | Noncoding RNA |
| Cullin-3 | Promotes Sleep | Promotes Rebound Following Deprivation | 2011, 2012 | 22196332, 23055946 | Cellular Homeostasis | Protein Degradation |
| Nedd8 ubiquitin like modifier | Promotes Sleep | ND | 2011 | 22196332 | Cellular Homeostasis | Protein Degradation |
| Homer | Promotes Sleep | None | 2012, 2019 | 22532843, 31418019 | Neural Signalling | Glutamatergic Signaling |
| insomniac | Promotes Sleep | Promotes Rebound Following Deprivation | 2012 | 23055946 | Cellular Homeostasis | Protein Degradation |
| basket | Promotes Sleep | None | 2011 | 22197814 | Gene Regulation | Kinase Signaling, Cell shape Regulation, Stress Response |
| regulator of cyclin A1 | Promotes Sleep | ND | 2012 | 22461610 | Cellular Homeostasis | Cell Cycle Control |
| cyclin A | Promotes Sleep | Promotes Rebound Following Deprivation | 2012 | 22461610 | Cellular Homeostasis | Cell Cycle Control |
| Sulfonylurea receptor | Promotes Sleep | ND | 2013 | 22105623 | Cellular Homeostasis | Potasium Channel, Heart Development |
| Vesicular monoamine transporter | Promotes Wakefulness | None | 2013 | 23658190 | Neural Signalling | Aminergic Signaling |
| Histidine decarboxylase | Promotes Wakefulness | ND | 2013 | 23844178 | Neural Signalling | Histamine Signaling |
| Histamine-gated chloride channel subunit 1 | Promotes Wakefulness | ND | 2013 | 23844178 | Neural Signalling | Histamine Signaling |
| short neuropeptide F receptor | Promotes Wakefulness | ND | 2013 | 23796436 | Neural Signalling | Neuropeptide Signaling |
| Neuroligin 4 | Promotes Sleep | ND | 2013 | 24068821 | Neural Signalling | Cell Adhesion, Synaptic Development |
| SIFamide | Promotes Sleep | ND | 2014 | 24658384 | Neural Signalling | Neuropeptide Signaling |
| SIFamide receptor | Promotes Sleep | ND | 2014 | 24658384 | Neural Signalling | Neuropeptide Signaling |
| Sex peptide receptor | Promotes Sleep | Promotes Rebound Following Deprivation | 2014 | 25333796 | Neural Signalling | Neuropeptide Signaling |
| Myoinhibiting peptide precursor | Promotes Sleep | Promotes Rebound Following Deprivation | 2014 | 25333796 | Neural Signalling | Neuropeptide Signaling |
| Diuretic hormone 31 | Promotes Wakefulness | ND | 2014 | 25455031 | Neural Signalling | Neuropeptide Signaling |
| γ-aminobutyric acid transaminase | Promotes Wakefulness | ND | 2015 | 24637426 | Neural Signalling | GABAergic Signaling |
| NMDA receptor 1 | Promotes Sleep | ND | 2015, 2019 | 26023770, 31064979 | Neural Signalling | Glutamatergic Signaling |
| Insulin-like peptide 1 | Promotes Sleep | ND | 2015 | 25581915 | Neural Signalling | Insulin Signaling |
| Insulin-like peptide 2 | Promotes Sleep | ND | 2015 | 25581915 | Neural Signalling | Insulin Signaling |
| Insulin-like peptide 3 | Promotes Sleep | ND | 2015 | 25581915 | Neural Signalling | Insulin Signaling |
| Insulin-like peptide 5 | Promotes Sleep | ND | 2015, 2021 | 25581915, 34998032 | Neural Signalling | Insulin Signaling |
| Insulin-like peptide 6 | Promotes Sleep | ND | 2015 | 25581915 | Neural Signalling | Insulin Signaling |
| Insulin-like peptide 7 | Promotes Sleep | ND | 2015 | 25581915 | Neural Signalling | Insulin Signaling |
| Insulin-like receptor | Promotes Sleep | ND | 2015 | 25581915 | Neural Signalling | Insulin Signaling |
| Anaplastic lymphoma kinase | Promotes Wakefulness | None | 2015 | 26536237 | Neural Signalling | Receptor Tyrosine Kinase Signaling |
| Neurofibromin 1 | Promotes Sleep | ND | 2015, 2023 | 26536237, 37593040 | Cellular Homeostasis | G Protein Signaling |
| Ca2+-channel protein ?1 subunit T | Promotes Wakefulness | ND | 2015 | 26647714 | Neural Signalling | Calcium Channel |
| Adenosine deaminase acting on RNA | Promotes Wakefulness | None | 2016 | 26813350 | Gene Regulation | RNA Editing |
| Dopamine 1-like receptor 2 | Promotes Wakefulness | ND | 2016 | 27487216 | Neural Signalling | Aminergic Signaling |
| Shaker cognate b | Promotes Sleep | ND | 2016 | 27487216 | Neural Signalling | Potasium Channel |
| sandman | Promotes Wakefulness | ND | 2016 | 27487216 | Neural Signalling | Potasium Channel |
| Transcription factor AP-2 | Promotes Sleep | ND | 2016 | 27829368 | Gene Regulation | Transcription Factor |
| Neurexin 1 | Promotes Sleep | Promotes Rebound Following Deprivation | 2016 | 27905548 | Neural Signalling | Neuropeptide Signaling |
| Vesicular acetylcholine transporter | Promotes Sleep | ND | 2016 | 27905548 | Neural Signalling | Cholinergic Signaling |
| β-Amyloid precursor protein binding protein 1 | Promotes Wakefulness | ND | 2017 | 28314820 | Neural Signalling | Post-Translational Protein Modification |
| Excitatory amino acid transporter 1 | Promotes Sleep | ND | 2017 | 28314820 | Neural Signalling | Glutamatergic Signaling |
| Open rectifier K+ channel 1 | Promotes Sleep | ND | 2017 | 28682878 | Neural Signalling | Potasium Channel |
| rogdi | Promotes Sleep | Suppresses Rebound Following Deprivation | 2017 | 28900300 | Neural Signalling | GABAergic Signaling |
| Tryptophan hydroxylase neuronal | Promotes Sleep | Promotes Rebound Following Deprivation | 2017 | 28984573 | Neural Signalling | Aminergic Signaling |
| 5-hydroxytryptamine (serotonin) receptor 2B | Promotes Sleep | Promotes Rebound Following Deprivation | 2017 | 28984573 | Neural Signalling | Aminergic Signaling |
| F box and leucine-rich-repeat gene 4 | Promotes Wakefulness | ND | 2017 | 29174887 | Cellular Homeostasis | Protein Degradation |
| Jumonji domain containing 5 | Promotes Sleep | ND | 2018 | 29339751 | Gene Regulation | Chromatin Regulation |
| Jumonji domain containing 7 | Promotes Sleep | ND | 2018 | 29339751 | Gene Regulation | Chromatin Regulation |
| Nucleolar protein 66 | Promotes Sleep | ND | 2018 | 29339751 | Gene Regulation | Chromatin Regulation |
| Lysine demethylase 4B | Promotes Wakefulness | ND | 2018 | 29339751 | Gene Regulation | Chromatin Regulation |
| let7 | Promotes Sleep | None | 2018 | 29949763 | Gene Regulation | MicroRNA |
| mir-984 stem loop | Promotes Sleep | None | 2018 | 29949763 | Gene Regulation | MicroRNA |
| mir-986 stem loop | Promotes Sleep | None | 2018 | 29949763 | Gene Regulation | MicroRNA |
| mir-977 stem loop | Promotes Sleep | None | 2018 | 29949763 | Gene Regulation | MicroRNA |
| bantam | Promotes Sleep | None | 2018 | 29949763 | Gene Regulation | MicroRNA |
| mir-1003 stem loop | Promotes Sleep | None | 2018 | 29949763 | Gene Regulation | MicroRNA |
| bereft (a.k.a. miR263a) | Promotes Sleep | None | 2018 | 29949763 | Gene Regulation | MicroRNA |
| mir-190 stem loop | Promotes Sleep | Promotes Rebound Following Deprivation | 2018 | 29949763 | Gene Regulation | MicroRNA |
| mir-184 stem loop | Promotes Sleep | None | 2018 | 29949763 | Gene Regulation | MicroRNA |
| mir-955 stem loop | Promotes Sleep | Suppresses Rebound Following Deprivation | 2018 | 29949763 | Gene Regulation | MicroRNA |
| mir-956 stem loop | Promotes Sleep | Suppresses Rebound Following Deprivation | 2018 | 29949763 | Gene Regulation | MicroRNA |
| mir-2b-1 stem loop | Promotes Sleep | None | 2018 | 29949763 | Gene Regulation | MicroRNA |
| mir-981 stem loop | Promotes Sleep | None | 2018 | 29949763 | Gene Regulation | MicroRNA |
| mir-1013 stem loop | Promotes Sleep | None | 2018 | 29949763 | Gene Regulation | MicroRNA |
| mir-992 stem loop | Promotss Sleep | None | 2018 | 29949763 | Gene Regulation | MicroRNA |
| mir-281-2 stem loop | Promotes Sleep | None | 2018 | 29949763 | Gene Regulation | MicroRNA |
| mir-962 stem loop | Promotes Sleep | None | 2018 | 29949763 | Gene Regulation | MicroRNA |
| mir-972 stem loop | Promotes Wakefulness | None | 2018 | 29949763 | Gene Regulation | MicroRNA |
| mir-954 stem loop | Promotes Wakefulness | None | 2018 | 29949763 | Gene Regulation | MicroRNA |
| mir-275 stem loop | Promotes Wakefulness | None | 2018 | 29949763 | Gene Regulation | MicroRNA |
| mir-92a stem loop | Promotes Wakefulness | None | 2018 | 29949763 | Gene Regulation | MicroRNA |
| mir-306 stem loop | Promotes Wakefulness | None | 2018 | 29949763 | Gene Regulation | MicroRNA |
| mir-92b stem loop | Promotes Wakefulness | None | 2018 | 29949763 | Gene Regulation | MicroRNA |
| mir-305 stem loop | Promotes Wakefulness | None | 2018 | 29949763 | Gene Regulation | MicroRNA |
| mir-310 stem loop | Promotes Wakefulness | None | 2018 | 29949763 | Gene Regulation | MicroRNA |
| mir-281-1 stem loop | None | Suppresses Rebound Following Deprivation | 2018 | 29949763 | Gene Regulation | MicroRNA |
| mir-313 stem loop | None | Suppresses Rebound Following Deprivation | 2018 | 29949763 | Gene Regulation | MicroRNA |
| mir-318 stem loop | None | Suppresses Rebound Following Deprivation | 2018 | 29949763 | Gene Regulation | MicroRNA |
| mir-957 stem loop | None | Suppresses Rebound Following Deprivation | 2018 | 29949763 | Gene Regulation | MicroRNA |
| mir-308 stem loop | None | Suppresses Rebound Following Deprivation | 2018 | 29949763 | Gene Regulation | MicroRNA |
| mir-1014 stem loop | None | Suppresses Rebound Following Deprivation | 2018 | 29949763 | Gene Regulation | MicroRNA |
| Innexin 6 | Promotes Sleep | ND | 2018 | 30109983 | Neural Signalling | Gap Junctions |
| pudgy | Promotes Sleep | Promotes Rebound Following Deprivation | 2018 | 30186232 | Cellular Homeostasis | Lipid Metabolism |
| minidiscs | Promotes Wakefulness | ND | 2018 | 30016498 | Cellular Homeostasis | Amino Acid Transport |
| Juvenile hormone Inducible-21 | Promotes Wakefulness | ND | 2018 | 30016498 | Cellular Homeostasis | Amino Acid Transport |
| eiger | Promotes Sleep | Promotes Rebound Following Deprivation | 2018 | 30379810 | Cellular Homeostasis | Cytokine Signaling |
| wengen | Promotes Sleep | Promotes Rebound Following Deprivation | 2018 | 30379810 | Cellular Homeostasis | Cytokine Signaling |
| Phosphoribosylformylglycinamidine synthase (a.k.a. Ade2) | Promotes Sleep | None | 2018 | 30249751 | Cellular Homeostasis | Purine Biosynthesis |
| Excitatory amino acid transporter 2 | Promotes Wakefulness | None | 2018 | 30416062 | Cellular Homeostasis | Amino Acid Transport |
| Neurocalcin | Promotes Sleep | ND | 2019 | 30865587 | Neural Signalling | Calcium Signaling |
| Serine hydroxymethyl transferase | Promotes Sleep | ND | 2019 | 31064979 | Cellular Homeostasis | Amino Acid Synthesis |
| Serine racemase | Promotes Sleep | None | 2019 | 31064979 | Cellular Homeostasis | Amino Acid Synthesis |
| metabotropic GABA-B receptor subtype 3 | Promotes Wakefulness | ND | 2019 | 31313987 | Neural Signalling | GABAergic Signaling |
| L-threonine dehydrogenase | Promotes Wakefulness | ND | 2019 | 31313987 | Cellular Homeostasis | Amino Acid Catabolism |
| noktochor | Promotes Sleep | None | 2019 | 31353186 | Not Known | Likely Intercellular Signaling |
| metabotropic Glutamate Receptor | Promotes Sleep | None | 2019 | 31418019 | Neural Signalling | Glutamatergic Signaling |
| eukaryotic translation initiation factor 2 subunit alphaF-2α kinase | Promotes Sleep | ND | 2020 | 32169212 | Cellular Homeostasis | Kinase Signaling |
| unpaired 2 | Promotes Sleep | ND | 2020 | 32745077 | Cellular Homeostasis | Cytokine Signaling |
| mir-276a stem loop | Promotes Wakefulness | ND | 2021 | 33337563 | Gene Regulation | MicroRNA |
| stuxnet | Promotes Wakefulness | Suppresses Rebound Following Deprivation | 2021 | 33410264 | Cellular Homeostasis | Protein Degradation |
| Polycomb | Promotes Sleep | ND | 2021 | 33410264 | Gene Regulation | Chromatin Regulation |
| Octopamine β1 receptor | Promotes Wakefulness | Suppresses Rebound Following Deprivation | 2021 | 33410264 | Neural Signalling | Aminergic Signaling |
| Octopamine β2 receptor | Promotes Wakefulness | Suppresses Rebound Following Deprivation | 2021 | 33410264 | Neural Signalling | Aminergic Signaling |
| Octopamine β3 receptor | Promotes Wakefulness | Suppresses Rebound Following Deprivation | 2021 | 33410264 | Neural Signalling | Aminergic Signaling |
| blue cheese | Promotes Wakefulness | ND | 2021 | 34085929 | Cellular Homeostasis | Vesicle Trafficking |
| Autophagy-related 1 | Promotes Wakefulness | ND | 2021 | 34085929 | Cellular Homeostasis | Autophagy |
| Heat shock protein 70 cognate 3 (a.k.a., BiP) | Promotes Wakefulness | ND | 2021 | 34085929 | Cellular Homeostasis | Endoplasmic Reticulum Chaperone |
| Autophagy-related 10 | Promotes Wakefulness | ND | 2021 | 34085929 | Cellular Homeostasis | Autophagy |
| Autophagy-related 8b | Promotes Wakefulness | ND | 2021 | 34085929 | Cellular Homeostasis | Autophagy |
| Autophagy-related 7 | Promotes Wakefulness | ND | 2021 | 34085929 | Cellular Homeostasis | Autophagy |
| Autophagy-related 12 | Promotes Wakefulness | ND | 2021 | 34085929 | Cellular Homeostasis | Autophagy |
| Another Drosophila Unc-51-like kinase | Promotes Wakefulness | ND | 2021 | 34085929 | Cellular Homeostasis | Autophagy |
| Atf6 | Promotes Wakefulness | ND | 2021 | 34085929 | Gene Regulation | Transcription Factor |
| Dram | Promotes Wakefulness | ND | 2021 | 34085929 | Cellular Homeostasis | Autophagy |
| wacky | Promotes Wakefulness | ND | 2021 | 34085929 | Cellular Homeostasis | Autophagy |
| kismet | Promotes Sleep | ND | 2021 | 34088660 | Gene Regulation | Chromatin Regulation |
| cacophony | Promotes Sleep | ND | 2021 | 34015490 | Neural Signalling | Calcium Channel |
| nicotinic Acetylcholine Receptor α2 | Promotes Sleep | ND | 2021 | 33493349 | Neural Signalling | Cholinergic Signaling |
| nicotinic Acetylcholine Receptor β2 | Promotes Sleep | ND | 2021 | 33493349 | Neural Signalling | Cholinergic Signaling |
| nicotinic Acetylcholine Receptor α1 | Promotes Sleep | ND | 2021 | 33493349 | Neural Signalling | Cholinergic Signaling |
| nicotinic Acetylcholine Receptor β1 | Promotes Sleep | ND | 2021 | 33493349 | Neural Signalling | Cholinergic Signaling |
| nicotinic Acetylcholine Receptor α5 | Promotes Wakefulness | ND | 2021 | 33493349 | Neural Signalling | Cholinergic Signaling |
| uncoordinated 79 | Promotes Wakefulness | ND | 2021 | 34849820 | Neural Signalling | Sodium Channel Subunit |
| Methoprene-tolerant | Promotes Wakefulness | ND | 2021 | 34376377 | Gene Regulation | Juvenile Hormone Receptor |
| Mesencephalic astrocyte-derived neurotrophic factor | Promotes Sleep | ND | 2021 | 33666288 | Cellular Homeostasis | Neurotrophic Factor |
| 14-3-3ε | Promotes Sleep | ND | 2021 | 34575915 | Cellular Homeostasis | Second Messenger Signaling |
| Hugin | Promotes Sleep | Suppresses Rebound Following Deprivation | 2021 | 34782479 | Neural Signalling | Neuropeptide Signaling |
| rumpel | Promotes Wakefulness | ND | 2021 | 34897385 | Cellular Homeostasis | Solute/Sodium Symporter |
| D-amino acid oxidase 1 | Promotes Wakefulness | ND | 2021 | 34922200 | Cellular Homeostasis | Amino Acid Catabolism |
| GABA transporter | Promotes Wakefulness | Promotes Rebound Following Deprivation | 2022 | 35303417 | Neural Signalling | GABAergic Signaling |
| Lipophorin receptor 1 | Promotes Wakefulness | ND | 2022 | 36071487 | Cellular Homeostasis | Lipid Uptake |
| Lipophorin receptor 2 | Promotes Wakefulness | ND | 2022 | 36071487 | Cellular Homeostasis | Lipid Uptake |
| Dab adaptor protein (a.k.a.disabled) | Promotes Wakefulness | ND | 2022 | 36071487 | Cellular Homeostasis | Tyrosine Kinase Signaling |
| Serotonin transporter | Promotes Wakefulness | ND | 2022 | 36409783 | Neural Signalling | Aminergic Signaling |
| U snoRNA host gene 4 | Promotes Wakefulness | ND | 2022 | 36451091 | Gene Regulation | Noncoding RNA |
| Desaturase 1 | Promotes Wakefulness | ND | 2022 | 35199930 | Pheromone Signalling | Cuticular Hydrocarbon Synthesis |
| Cytochrome P450 4g1 | Promotes Wakefulness | ND | 2022 | 35199930 | Pheromone Signalling | Cuticular Hydrocarbon Synthesis |
| pickpocket 23 | Promotes Wakefulness | ND | 2022 | 35199930 | Pheromone Signalling | Pheromone Perception |
| pickpocket 29 | Promotes Wakefulness | ND | 2022 | 35199930 | Pheromone Signalling | Pheromone Perception |
| Connectin | Promotes Sleep | ND | 2023 | 36608130 | Cellular Homeostasis | Cell Adhesion, Synaptic Development |
| daughterless | Promotes Sleep | ND | 2023 | 36608130 | Gene Regulation | Transcription Factor |
| homothorax | Promotes Sleep | ND | 2023 | 36608130 | Gene Regulation | Transcription Factor |
| G protein beta subunit 13F | Promotes Wakefulness | ND | 2023 | 36608130 | Cellular Homeostasis | G Protein Signaling |
| twister | Promotes Wakefulness | ND | 2023 | 36608130 | Gene Regulation | Alternative Splicing |
| Phosphatidylinositol glycan anchor biosynthesis class Q | Promotes Wakefulness | ND | 2023 | 36608130 | Cellular Homeostasis | Membrane Anchor |
| Phosphatidylinositol glycan anchor biosynthesis class Z | Promotes Wakefulness | None | 2023 | 36608130 | Cellular Homeostasis | Membrane Anchor |
| Phosphatidylinositol glycan anchor biosynthesis class O | Promotes Wakefulness | ND | 2023 | 36608130 | Cellular Homeostasis | Membrane Anchor |
| Phosphatidylinositol glycan anchor biosynthesis class C | Promotes Wakefulness | ND | 2023 | 36608130 | Cellular Homeostasis | Membrane Anchor |
| Phosphatidylinositol glycan anchor biosynthesis class G | Promotes Wakefulness | ND | 2023 | 36608130 | Cellular Homeostasis | Membrane Anchor |
| Phosphatidylinositol glycan anchor biosynthesis class M | Promotes Wakefulness | ND | 2023 | 36608130 | Cellular Homeostasis | Membrane Anchor |
| Ecdysone receptor | Promotes Sleep | ND | 2023 | 36719183 | Gene Regulation | Nuclear Hormone Receptor |
| Ecdysone-induced protein 75B | Promotes Sleep | ND | 2023 | 36719183 | Gene Regulation | Nuclear Hormone Receptor |
| ftz transcription factor 1 | Promotes Sleep | ND | 2023 | 36719183 | Gene Regulation | Likely Nuclear Hormone Receptor |
| Hormone receptor 3 | Promotes Sleep | ND | 2023 | 36719183 | Gene Regulation | Nuclear Hormone Receptor |
| retinal degeneration B | Promotes Sleep | ND | 2023 | 36586155 | Cellular Homeostasis | G Protein Signaling |
| Cytoplasmic FMR1 interacting protein | Promotes Sleep | Promotes Rebound Following Deprivation | 2023 | 36808152 | Cellular Homeostasis | Cytoskeleton Regulation |
| allnighter | Promotes Sleep | ND | 2023 | 37217484 | Cellular Homeostasis | Pseudokinase |
| ebony | Promotes Wakefulness | ND | 2023 | 37369755 | Neural Signalling | Aminergic Signalling, Cuticle Formation |
| Pallidin | Promotes Sleep | ND | 2023 | 37682712 | Cellular Homeostasis | Lysosome Biogenesis |
| Biogenesis of lysosome-related organelles complex 1, subunit 1 | Promotes Sleep | ND | 2023 | 37682712 | Cellular Homeostasis | Lysosome Biogenesis |
| Biogenesis of lysosome-related organelles complex 1, subunit 2 | Promotes Sleep | ND | 2023 | 37682712 | Cellular Homeostasis | Lysosome Biogenesis |
| Dysbindin | Promotes Sleep | ND | 2023 | 37682712 | Cellular Homeostasis | Lysosome Biogenesis |
| Juvenile hormone Inducible-21 | Promotes Sleep | ND | 2023 | 37682712 | Cellular Homeostasis | Amino Acid Transport |
| minidiscs | Promotes Sleep | ND | 2023 | 37682712 | Cellular Homeostasis | Amino Acid Transport |
| raptor | Promotes Sleep | ND | 2023 | 37682712 | Cellular Homeostasis | TOR Signaling |
| mechanistic Target of rapamycin | Promotes Sleep | ND | 2023 | 37682712 | Cellular Homeostasis | TOR Signaling |
| moody | Promotes Sleep | ND | 2023 | 37831742 | Cellular Homeostasis | Blood Brain Barrier |
| G protein alpha subunit o | Promotes Sleep | ND | 2023 | 37831742 | Cellular Homeostasis | G Protein Signaling |
| locomotion defects | Promotes Sleep | ND | 2023 | 37831742 | Cellular Homeostasis | G Protein Signaling |
| Lachesin | Promotes Sleep | ND | 2023 | 37831742 | Cellular Homeostasis | Septate Junctions |
| Neuroglian | Promotes Sleep | ND | 2023 | 37831742 | Cellular Homeostasis | Cell Adhesion, Synaptic Development |
| Protein kinase, cAMP-dependent, catalytic subunit 1 | Promotes Sleep | ND | 2023 | 37831742 | Cellular Homeostasis | Kinase Signaling |

**Table 3:**

| Gene Name | Role in Baseline Sleep | Role in Sleep Rebound | Year | PMID | General Mechanism | Specific Mechanism |
| --- | --- | --- | --- | --- | --- | --- |
| Tryptophan hydroxylase neuronal | Promotes Sleep | Promotes Rebound Following Deprivation | 2017 | 28984573 | Neural Signalling | Aminergic Signaling |
| Octopamine β3 receptor | Promotes Wakefulness | Suppresses Rebound Following Deprivation | 2021 | 33410264 | Neural Signalling | Aminergic Signaling |
| Octopamine β2 receptor | Promotes Wakefulness | Suppresses Rebound Following Deprivation | 2021 | 33410264 | Neural Signalling | Aminergic Signaling |
| Octopamine β1 receptor | Promotes Wakefulness | Suppresses Rebound Following Deprivation | 2021 | 33410264 | Neural Signalling | Aminergic Signaling |
| fumin | Promotes Sleep | Promotes Rebound Following Deprivation | 2005, 2008 | 16093388, 18457233 | Neural Signalling | Aminergic Signaling |
| 5-hydroxytryptamine (serotonin) receptor 2B | Promotes Sleep | Promotes Rebound Following Deprivation | 2017 | 28984573 | Neural Signalling | Aminergic Signaling |
| cAMP Response Element Binding Protein (CREB) | Promotes Wakefulness | Suppresses Rebound Following Deprivation | 2001 | 11687816 | Cellular Homeostasis | cAMP Signaling |
| cyclin A | Promotes Sleep | Promotes Rebound Following Deprivation | 2012 | 22461610 | Cellular Homeostasis | Cell Cycle Control |
| wengen | Promotes Sleep | Promotes Rebound Following Deprivation | 2018 | 30379810 | Cellular Homeostasis | Cytokine Signaling |
| eiger | Promotes Sleep | Promotes Rebound Following Deprivation | 2018 | 30379810 | Cellular Homeostasis | Cytokine Signaling |
| Cytoplasmic FMR1 interacting protein | Promotes Sleep | Promotes Rebound Following Deprivation | 2023 | 36808152 | Cellular Homeostasis | Cytoskeleton Regulation |
| rhomboid | Promotes Sleep | Promotes Rebound Following Deprivation | 2007 | 17694052 | Cellular Homeostasis | Growth Factor Signaling |
| pudgy | Promotes Sleep | Promotes Rebound Following Deprivation | 2018 | 30186232 | Cellular Homeostasis | Lipid Metabolism |
| mir-190 stem loop | Promotes Sleep | Promotes Rebound Following Deprivation | 2018 | 29949763 | Gene Regulation | MicroRNA |
| Sex peptide receptor | Promotes Sleep | Promotes Rebound Following Deprivation | 2014 | 25333796 | Neural Signalling | Neuropeptide Signaling |
| Neurexin 1 | Promotes Sleep | Promotes Rebound Following Deprivation | 2016 | 27905548 | Neural Signalling | Neuropeptide Signaling |
| Myoinhibiting peptide precursor | Promotes Sleep | Promotes Rebound Following Deprivation | 2014 | 25333796 | Neural Signalling | Neuropeptide Signaling |
| yellow-achaete intergenic RNA | Promotes Sleep | Promotes Rebound Following Deprivation | 2011 | 21775470 | Gene Regulation | Noncoding RNA |
| Ecdysone receptor | Promotes Sleep | Promotes Rebound Following Deprivation | 2010 | 20215472 | Gene Regulation | Nuclear Hormone Receptor, Steroid Signaling |
| sleepless (a.k.a., quiver) | Promotes Sleep | Promotes Rebound Following Deprivation | 2008 | 18635795 | Neural Signalling | Potassium Channel |
| stuxnet | Promotes Wakefulness | Suppresses Rebound Following Deprivation | 2021 | 33410264 | Cellular Homeostasis | Protein Degradation |
| insomniac | Promotes Sleep | Promotes Rebound Following Deprivation | 2012 | 23055946 | Cellular Homeostasis | Protein Degradation |
| Cullin-3 | Promotes Sleep | Promotes Rebound Following Deprivation | 2011, 2012 | 22196332, 23055946 | Cellular Homeostasis | Protein Degradation |
